# Long-term prediction of prostate cancer diagnosis and death using PSA and obesity related anthropometrics at early middle age: data from the malmö preventive project

**DOI:** 10.18632/oncotarget.22981

**Published:** 2017-12-05

**Authors:** Melissa J. Assel, Axel Gerdtsson, Daniel L.J. Thorek, Sigrid V. Carlsson, Johan Malm, Peter T. Scardino, Andrew Vickers, Hans Lilja, David Ulmert

**Affiliations:** ^1^ Department of Epidemiology and Biostatistics, Memorial Sloan Kettering Cancer Center, New York, NY, USA; ^2^ Department of Clinical Sciences, Lund University, Skåne University Hospital, Malmö, Sweden; ^3^ Department of Translational Medicine, Lund University, Malmö, Sweden; ^4^ Division of Nuclear Medicine and Molecular Imaging, Department of Radiology and Radiological Science, Sidney Kimmel Comprehensive Cancer Center, Johns Hopkins School of Medicine, Baltimore, MD, USA; ^5^ Urology Service, Department of Surgery, Memorial Sloan Kettering Cancer Center, New York, NY, USA; ^6^ Institute of Clinical Sciences, Sahlgrenska Academy at Gothenburg University, Gothenburg, Sweden; ^7^ Departments of Laboratory Medicine and Medicine, Memorial Sloan Kettering Cancer Center, New York, NY, USA; ^8^ Nuffield Department of Surgical Sciences, University of Oxford, Oxford, UK; ^9^ Molecular Pharmacology Program, Sloan Kettering Institute, Memorial Sloan Kettering Cancer Center, New York, NY, USA

**Keywords:** PSA, obesity, BMI, weight, prostate cancer

## Abstract

**Objectives:**

To evaluate whether anthropometric parameters add to PSA measurements in middle-aged men for risk assessment of prostate cancer (PCa) diagnosis and death.

**Results:**

After adjusting for PSA, both BMI and weight were significantly associated with an increased risk of PCa death with the odds of a death corresponding to a 10 kg/m2 or 10 kg increase being 1.58 (95% CI 1.10, 2.28; *p* = 0.013) and 1.14 (95% CI 1.02, 1.26; *p* = 0.016) times greater, respectively. AUCs did not meaningfully increase with the addition of weight or BMI to prediction models including PSA.

**Materials and Methods:**

In 1974 to 1986, 22,444 Swedish men aged 44 to 50 enrolled in Malmö Preventive Project, Sweden, and provided blood samples and anthropometric data. Rates of PSA screening in the cohort were very low. Documentation of PCa diagnosis and disease-specific death up to 2014 was retrieved through national registries. Among men with anthropometric measurements available at baseline, a total of 1692 men diagnosed with PCa were matched to 4190 controls, and 464 men who died of disease were matched to 1390 controls. Multivariable conditional logistic regression was used to determine whether diagnosis or death from PCa were associated with weight and body mass index (BMI) at adulthood after adjusting for PSA.

**Conclusions:**

Men with higher BMI and weight at early middle age have an increased risk of PCa diagnosis and death after adjusting for PSA. However, in a multi-variable numerical statistical model, BMI and weight do not importantly improve the predictive accuracy of PSA. Risk-stratification of screening should be based on PSA without reference to anthropometrics.

## INTRODUCTION

In the vast majority of cases, prostate cancer (PCa) is diagnosed in the elderly, which is a result of the slow pathogenic process of the disease. Given the combination of wide prevalence, and slow rate of progression [1, 2], we and others have reasoned that there is also a considerable therapeutic window in which lifestyle choices may be modified to augment disease prevention. To act on this reasoning, the field must be adequately informed through unbiased empirical measures of anthropometric factors and risk of PCa incidence and mortality.

Several studies have shown that weight and Body Mass Index (BMI) impact PCa risk estimates [3]. Using anthropometric data collected from men who participated in a large, representative population-based preventive project conducted in Malmö, Sweden (MPP) in 1974 to 1986, we have previously shown that BMI and weight during early middle age, but not earlier in life (birth to adolescence), predict distant metastasis or death from the disease [4]. This raises the question of whether obesity related anthropometrics could be used to risk stratify screening at early middle age. Within the MPP study we have total PSA measurements assayed in frozen plasma samples collected in conjuncture with the BMI and weight measurement. We have previously reported that PSA levels at early middle age are strongly predictive of the risk of death or distant metastasis from prostate cancer several decades later, with high predictive accuracy (area under the curve, AUCs up to 0.90) in this MPP cohort [5–8]. In the current study our goal was to investigate whether BMI or weight adds to PSA in terms of aiding in risk stratification. Additionally, we wished to repeat our previous analysis investigating the association between BMI and weight at early middle age and prostate cancer outcomes with additional follow-up.

## RESULTS

Participant characteristics of cases and controls are given in Table 1. There were 1692 men diagnosed with PCa before December 31st, 2014 who had PSA and anthropometric data available. The median age at diagnosis of the cohort was 69 years. The median follow up time from screening date for men not diagnosed with prostate cancer was 34 years (IQR 25, 35). As displayed in Table 1, 75% of patients had a BMI between 23 kg/m^2^ and 27 kg/m^2^. Approximately 6% (312) of the cohort would be considered obese according to the Centers for Disease Control (CDC) guidelines with a BMI greater than or equal to 30 kg/m^2^.

**Table 1:** Patient characteristics

Characteristics	Cases (*N* = 1692; 32%)	Controls (*N* = 3636; 68%)
Age at blood draw	47 (45, 48)	47 (46, 48)
Weight (kg)	77 (71, 85)	76 (70, 83)
BMI (kg/m^2^)^*^	24 (23, 27)	24 (23, 27)
Total PSA (ng/mL)	1.03 (0.64, 1.90)	0.59 (0.37, 0.95)

Data are presented as median (IQR). Cases also used as controls are represented only in the cases group. ^*^BMI = body mass index.

**Figure 1:**
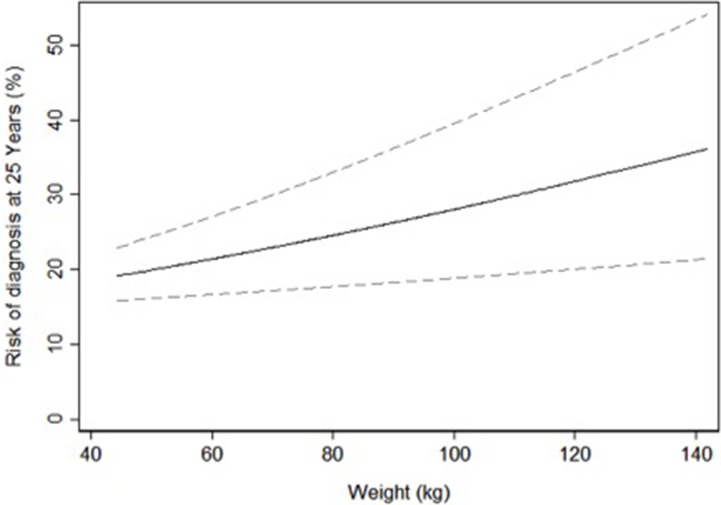
Univariable predicted risk of prostate cancer diagnosis 25 years after screening by weight at middle age

**Figure 2:**
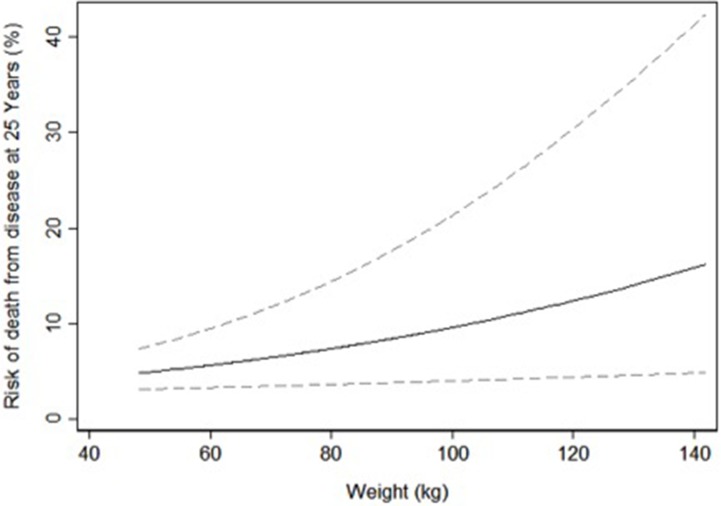
Univariable predicted risk of death from prostate cancer 25 years after screening by weight at middle age

**Figure 3:**
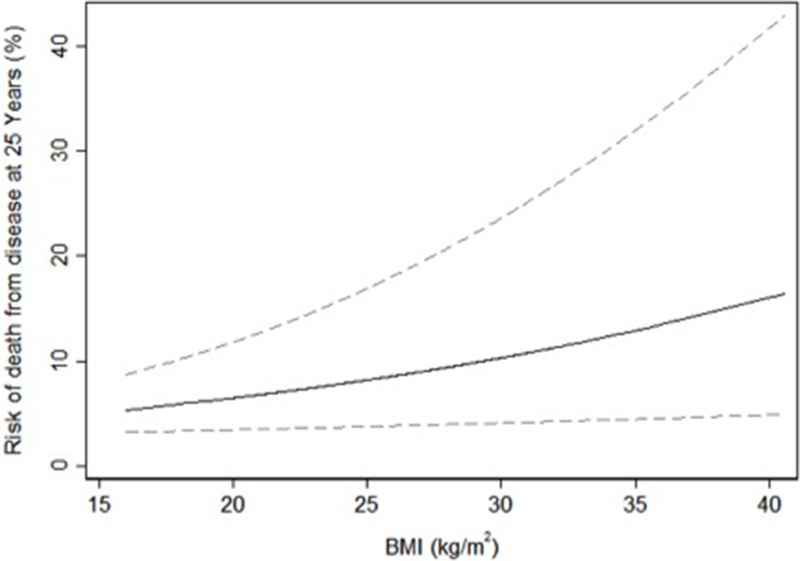
Univariable predicted risk of death from prostate cancer 25 years after screening by body mass index at middle age

Despite adding almost a decade of follow-up the results corresponding to the unadjusted analysis replicating our prior work were nearly identical to the multivariable analyses. The univariable predicted risk of PCa diagnosis by weight at middle age is shown in Figure 1 and the univariable predicted risk of PCa death by weight and BMI at middle age in Figures 2 and 3.

**Figure 4:**
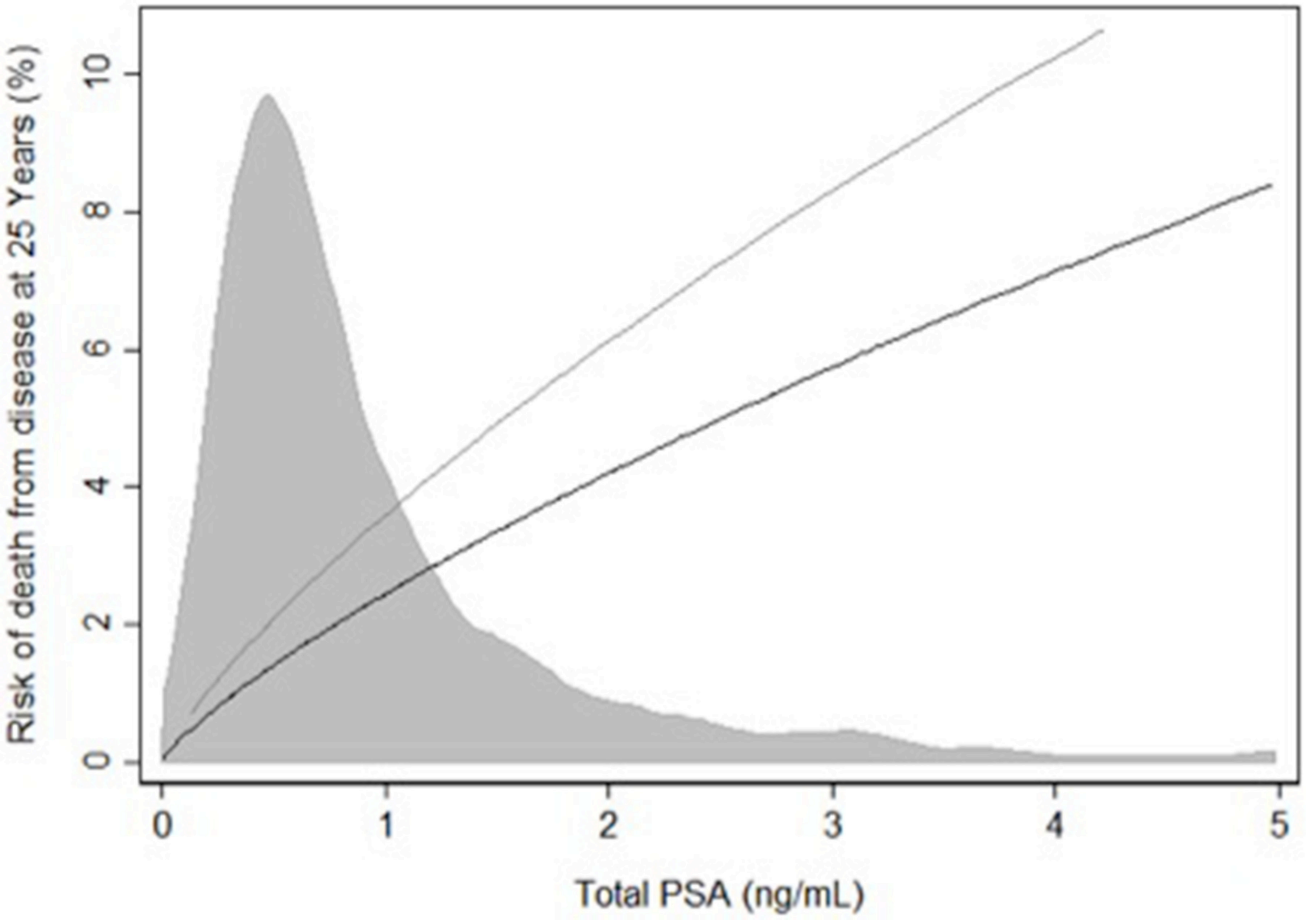
Predicted risk of death from prostate cancer 25 years after screening by total PSA (ng/mL) at screening separately for those with a BMI greater than or equal to 30 and for those with a BMI less than 30 Shaded region represents the distribution of total PSA. Grey Line: BMI ≥ 30. Black Line: < 30.

Measures of BMI and weight at middle-age were significantly associated with an increased risk of PCa death after adjustment for PSA. Table 2 shows that the odds of a death corresponding to a 10 kg/m^2^ or 10 kg increase were 1.58 and 1.14 times greater, respectively (*p*-values=0.013 and 0.016). Furthermore, weight at middle age was associated with a PCa diagnosis with an odds ratio of 1.09 per a 10 kg increase (Table 2, *p* = 0.002). Interactions between BMI or weight and PSA were non-significant (all *p*-values ≥ 0.3), as were the interactions between BMI or weight and age at health screening (all *p*-values ≥ 0.074). Although the interactions between BMI or weight and PSA were not significant, the predicted risk of PCa death by total PSA and obesity status (BMI ≤ 30 vs BMI > 30) was displayed in Figure 4. The increase in predicted risk of PCa death associated with obesity is less than 1% for those with a PSA less than 0.2 ng/mL, compared to a more than 3% higher risk at a total PSA above 4 ng/mL.

**Table 2:** Multivariable conditional logistic regression results corresponding to the test of association between the anthropometric predictor listed and the outcomes after adjusting for PSA

Model adjusted for PSA						
	BMI at Middle Age			Weight at Middle Age		
Outcome	OR^*^	95% CI	*p*-value	OR^†^	95% CI	*p*-value
PC Diagnosis	1.21	0.99, 1.47	0.063	1.09	1.03, 1.16	0.002
PC Death	1.58	1.10, 2.28	0.013	1.14	1.02, 1.26	0.016

^*^Odds ratio represents a 10 kg/m^2^ increase

^**†**^Odds ratio represents a 10 kg increase.

The addition of weight at middle age or BMI to prediction models based on total PSA-level at baseline do not meaningfully increase the calculated AUCs (Table 3). Although adult weight and BMI were significantly associated with PCa death and, or, diagnosis, respectively, the discriminative ability of the model did not meaningfully improve with these anthropometric additions. The largest observed change was an increase in AUC for the model predicting PCa death, where the addition of BMI to PSA increased AUC by 0.002 (Table 3). The incremental increase in discrimination with the addition of BMI did not meaningfully change based on the sensitivity analysis. However, the largest BMI in the re-sampled dataset was 45, which does not fully represent the range of BMIs of men in the United States.

**Table 3:** AUCs for models including PSA

Outcome	PSA	PSA + BMI	PSA + Weight
PC Diagnosis	0.689 (0.674, 0.703)	0.690 (0.675, 0.705)	0.689 (0.674, 0.703)
PC Death	0.695 (0.667, 0.723)	0.697 (0.669, 0.725)	0.696 (0.668, 0.725)

## DISCUSSION

We have demonstrated that the increased risk of PCa diagnosis and death related to excess weight and obesity at early-middle age was independent of PSA, implying that obese men are at higher risk regardless of PSA level. Our results show that a PSA level in an obese man is related to a higher risk compared to a non-obese. However, our analysis also show that combining a PSA measurement with an obesity related anthropometric measurement in a multi-variable numerical statistical model did not meaningfully aid in risk prediction compared to PSA alone. When we investigated the incremental value in a patient population with a similar distribution of BMI as the United States we also did not find BMI meaningfully aided in risk prediction. However, we did not account for highly obese people so we cannot exclude the possible utility of BMI in a setting with a larger range of BMIs. We further investigated whether the relationship between PSA and risk is modified by weight or BMI. We did not find that this was the case as there was no evidence of an interaction between PCa outcomes and weight or BMI.

Concomitant with the global increase in PCa diagnosis and the adoption of PSA screening, a dramatic increase in the prevalence of obesity has occurred. It is widely recognized that the associations between obesity and PCa risk are complex. The MPP study is a cardiovascular risk factor study and obese and overweight participants were counseled on the risks of obesity [16]. In addition to being counseled about their risk of cardiovascular disease, men who are obese or overweight should also be informed that increased BMI and weight leads to an increased risk of aggressive and fatal PCa [17–20].

To the best of our knowledge, this is a completely novel study in the field of oncology utilizing a tissue specific biomarker and whole body morphological measurements at early middle age with up to four decades of follow-up. Given the extensive follow-up and historically low PSA screening rates in this cohort, we are able to assess the added value PSA and weight or BMI in addition to PSA in predicting PCa diagnosis and death. Our data for analysis consisted of measurements of PSA from frozen blood plasma, weight, and BMI obtained at study enrollment during 1974–1986, which we linked to information on prostate cancer diagnosis and cause of death through to December 31st 2014. Published estimates of rates of PSA screening in Sweden indicate that testing was extremely rare before 1995. Between 1995 and 2000, the proportion of men aged 55–69 who received a PSA test increased steadily from < 1% to 10%. This screening rate had remained constant until 2005, after which it fell [21]. Apart from the advantageously low screening rate, our study utilized a dataset distinguished by its size, an impressive participation rate (74% of the target population), length of follow-up, inclusion of men at ages relevant to decisions about screening, and use of carefully ascertained metastasis or death from prostate cancer as an endpoint. Together, these reports have shown that a PSA measurement at early middle age is a very strong risk predictor at early age for clinically relevant and metastatic PCa, including disease related deaths [5–8].

Use of the MPP cohort to study the relationship between anthropometrics throughout life and clinically detected PCa has revealed that body weight and BMI at early-middle age, but not earlier in life, are associated with aggressive PCa [4]. In the current study, we corroborated these results based on a decade longer follow-up. Recently, similar age related observations were confirmed by data from the PLCO study, albeit using self-reported anthropometrics [20].

Our study was limited by the range of BMI values, no patients within our study cohort had a BMI ≥ 45. Comparatively, 0.55% of the 2010 US population has a BMI > 50 [22]. These results may not be generalizable to men in the United States or other countries with high obesity rates as we may underestimate the influence of very high BMI or weight for very obese men. Moreover, we may have underestimated the improvement in discrimination afforded by BMI. This is because discrimination is strongly influenced by heterogeneity: a variable cannot discriminate if it does not vary.

In conclusion, our findings indicate that there is an important relationship between BMI and weight at early middle age and PCa outcomes; obese men have a higher PCa risk regardless of PSA level and could therefore benefit from more extensive screening. However, we did not find that a multi-variable model combining BMI, or weight, with PSA blood biomarkers increased risk stratification compared to PSA alone. Risk-stratification of screening should be based on PSA without reference to BMI.

## MATERIALS AND METHODS

### Study population

The study participants were males enrolled in the Malmö Preventive Project (MPP). The MPP was a prospective preventive study begun in 1974, inviting all men born from 1921–1949 living in Malmö to receive a baseline evaluation including a questionnaire, physical examination and blood sampling. In total 22,444 men enrolled, representing 74% of eligible participants [9]. A Personal Identity Number (PIN), unique for every Swedish citizen, was used for tracking and merging of data from national cancer registries [10].

### Anthropometric measurements and PSA at middle age

Weight and height were recorded at baseline as part of participation in the MPP study. Total PSA was assayed in EDTA anticoagulated blood plasma stored at −20°C. These measurements were as previously reported [11] and performed by Dr Lilja’s laboratory at the Wallenberg Research Laboratories at Lund University in Malmö using the dual label DELFIA Prostatus total/free PSA assay (Perkin-Elmer, Turku, Finland), calibrated in accordance to the change implemented in 2004 to reflect the World Health Organization 96/670 calibration. All measures were conducted blind to outcome.

### Endpoint retrieval and matching of cases and controls

Our main endpoints were diagnosis and death due to PCa. By December 31, 2014, 2380 males in the MPP had been diagnosed with PCa according to the Swedish Cancer Registry. The systematic collection of data for the Registry was very high; including an estimated 95.4% of prostate cancers in 1978 [12]. A total of 49 indolent PCa cases diagnosed at autopsy were not considered cases and were included in the control selection process. We excluded 639 cases for which either no relevant weight, BMI information or blood sample for PSA was available, leaving us with 1692 unique cases. We aimed to complete a 1:3 matching: cases were matched to 4190 controls without a PCa-diagnosis at the date when the index case was diagnosed, and who were born within a year of the index case. For the analysis of PCa diagnosis, we have 5882 men in total as some cases were also used as controls (e.g. a participant diagnosed in 1995 can serve as a control for a participant diagnosed in 1990). Using similar criteria as for the control set – age at early middle age blood draw ± 1 year, alive and free of the index event at the date of diagnosis of that event – we performed a second matching for the outcome of PCa death to obtain 464 death cases (PCa deaths) and 1390 controls (men were alive at the time of the index case’s death, irrespective of PCa status). The assessment and ascertainment of PCa death was performed in accordance to previous reports [13, 14]. In brief, cause of death was obtained from the Swedish National Cause of Death Registry, but for all men who died subsequent to a diagnosis of prostate cancer, cause of death was by chart review.

### Ethical considerations

This study was approved by the Ethical board of Lund University. DNR 2010/45 and 2007/268.

### Statistical analysis

In order to assess whether weight or BMI at early middle age adds the predictiveness of PSA alone, we tested the association between these anthropometric measures and the risk of PCa diagnosis or death from PCa using multivariable conditional logistic regression. Because the relationship between anthopometric data (BMI and weight) and the PCa outcomes may not be linear, we tested for non-linearity using restricted cubic splines with knots at the tertiles. We did not find evidence of non-linearity for weight or BMI at middle age for any outcome after adjusting for log-transformed PSA values. The possibility of splines was investigated for PSA but we did not find sufficient evidence of non-linearity after transformation.

We evaluated the increment in predictive accuracy when the anthropometric data were added to the “base” multivariable conditional logistic regression models that included PSA. Discriminative accuracy was given as the area under the receiver operating characteristic curve (AUC). The AUCs corresponding to the multivariable models were corrected for overfit using 10-fold cross-validation.

In addition to predictive accuracy, we wished to assess whether the risk of PCa outcomes had a steeper increase by PSA among patients with a higher BMI. Thus, we included an interaction term for PSA and BMI in a multivariable model including PSA and BMI. Similarly, we wished to assess whether the risk of PCa outcomes had a steeper increase by age at assessment among patients with a higher weight or BMI and we included an interaction term for age and either BMI or weight in multivariable models including PSA, BMI or weight and age at screening.

Because of the case control design, absolute risks are misestimated: the average risk for PCa diagnosis and death from PCa would be at 25% due to the 3:1 matching. To calculate absolute risk, we recalibrated with a Bayes factor, using the observed incidence of each endpoint in the full MPP cohort. Bootstrap resampling was used to estimate the 95% confidence intervals around those risks. Where the association between middle-age weight or middle age BMI and the outcomes of interest was statistically significant after adjusting for PSA, we plotted the risk of the outcome by adult weight or BMI at age 70.

Additionally, we wished to replicate our previous findings based on longer follow-up. We tested the association between BMI and weight at early middle age and the risk of PCa diagnosis and PCa death using univariable conditional logistic regression. As a sensitivity analysis we calculated the incremental increase in discrimination with the addition of BMI in a dataset generated through a weighted re-sampling of study participants based on the distribution of BMI in the United States [15] Prevalence of prostate cancer and death from prostate cancer were assigned according to risk within each BMI category. All analyses were conducted using Stata 12.0 (Stata Corp., College Station, TX). A *p*-value less than 0.05 was considered significant.
